# Anti-inflammatory activity of *Boletus aereus* polysaccharides: Involvement of digestion and gut microbiota fermentation

**DOI:** 10.1016/j.fochx.2023.101052

**Published:** 2023-12-09

**Authors:** Shixiang Wei, Luanfeng Wang, Xiaodie Chen, Yue Wang, Lingling Tong, Qianyun Han, Bo Ren, Dongsheng Guo

**Affiliations:** a School of Food Science and Pharmaceutical Engineering, Nanjing Normal University Nanjing 210023, China; b College of Food Science and Engineering, Nanjing University of Finance and Economics/Collaborative Innovation Center for Modern Grain Circulation and Safety, Nanjing 210023, China; c BIOSYST-MeBioS, Faculty of Bioscience Engineering, KU Leuven, Leuven 3000, Belgium; d College of Food Science and Nutritional Engineering, China Agricultural University, 17 Qinghua East Road, Beijing 100083, China

**Keywords:** *Boletus aereus*, Polysaccharides, *In vitro* digestion, Gut microbiota, Anti-inflammatory activity

## Abstract

*Boletus aereus*, an edible mushroom, has gained popularity as a medicinal and functional food. This study aimed to investigate the digestive characteristics of *B. aereus* polysaccharide (BAP) and its effects on gut microbiota. *In vitro* digestion results indicated partial degradation of BAP. Furthermore, the digested BAP displayed significantly enhanced antioxidant ability. The 16S rRNA sequencing data revealed that BAP positively influenced the abundance of *Phascolarctobacterium*, *Prevotella*, and *Bifidobacterium* in the gut microbiota. Additionally, BAP promoted the production of short-chain fatty acids (SCFAs). Metabolites of BAP utilized by the gut microbiota effectively reduced the concentration of TNF-α, IL-1β, and NO in an LPS-stimulated RAW 264.7 cell inflammation model. Mantel tests demonstrated a strong correlation among fermentation indicators, gut microbiome composition, SCFAs, and inflammatory cytokines. Overall, this research revealed the underlying digestive and fermentation mechanisms of BAP and provided new insights into the usage of edible mushroom polysaccharides in functional food.

## Highlights

• *Boletus aereus* polysaccharides (BAP) could be partially digested in gastrointestinal tract.
• BAP produced SCFAs and promoted the growth of beneficial gut bacteria.
• *In vitro* fermentation improved the anti-inflammatory activity of BAP.
• Mantel tests indicated fermentation indicators, gut microbiome, SCFAs, and inflammatory cytokine were closely related.

## Graphical abstract

**Figure:**
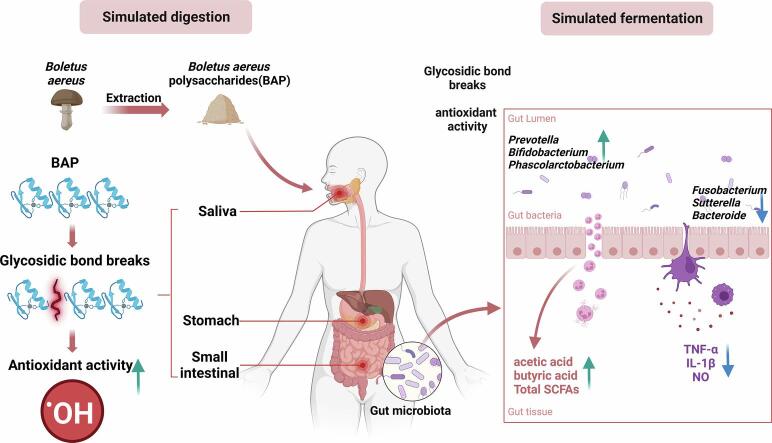

## 1 Introduction

Edible mushrooms, classified as macro fungi, exhibit notable pharmacological activities. Previous studies have highlighted the considerable potential of natural polysaccharides found in edible mushrooms as prebiotics (Hyder & Dutta, 2021). These polysaccharides offer unique physiological benefits, including immunomodulatory, antitumor, anti-inflammatory, antioxidant, and intestinal health-promoting properties (Maity et al., 2021). *Boletus aereus* is a familiar edible mushroom. Extensive research has demonstrated that *B. aereus* is not only rich in nutrients but also possesses remarkable biological activities; its polysaccharides, polyphenols, and proteins have been identified as the main active components, exhibiting antihyperglycemic and antioxidant properties (Kaewnarin et al., 2016). Furthermore, recent investigations have shown that *B. aereus* polysaccharides (BAPs) exhibit tumor growth inhibition and possess antitumor effects against S180 cells (Zheng et al., 2021). However, research on the digestion, fermentation, and prebiotic effects of BAP remains scarce.

*In vitro* digestion models and fermentation models can simulate the structural and activity changes of polysaccharides in the digestive system, which helps us to understand the digestive characteristics of polysaccharides and their effects on gut microbiota. This method is simple to perform and can evaluate the digestion of any food or nutrient and determine its impact on the microbiome. Natural polysaccharides are active, but they are difficult for cells to absorb and utilize directly due to their large relative molecular weights and complex structures. The biological activity of polysaccharides is fundamentally influenced by their structure. During digestion and *in vitro* fermentation, polysaccharides may undergo changes in their monosaccharide composition, relative molecular mass, glycosidic bond type, and bond configuration, leading to variations in their biological activity (Li et al., 2022). Studies on *Nostoc commune* Vauch. polysaccharides (NCVP) have demonstrated significant changes in monosaccharide composition after digestion, accompanied by an increase in antioxidant activity (Li et al., 2022). Furthermore, the polysaccharide from Chinese yam exhibited enhanced inhibition of inflammatory factors after digestion, with the emergence of small, loose fragments observed in the surface microstructure (Bai et al., 2023). These findings emphasize the importance of understanding the digestive characteristics of polysaccharides to elucidate structure–activity relationships.

Numerous studies have demonstrated that incompletely digested polysaccharides undergo further fermentation by the gut microbiota. Initially, specific primary degrading bacteria breakdown digestible polysaccharides into oligosaccharides, monosaccharides, and disaccharides. Subsequently, these polysaccharides are fermented, leading to the production of lactic acid and acetic acid. Secondary degrading bacteria then further ferment and degrade the primary degradation products of polysaccharides, resulting in the generation of final metabolites (Zhao et al., 2018). The gut microbiota plays a crucial role in utilizing various polysaccharides. For instance, the gut microbiota utilizes four different types of polysaccharides found in Tibetan tea to synthesize *n*-butyric acid, acetic acid, and propionic acid (Tan et al., 2023). Moreover, polysaccharides can modulate the abundance and composition of the gut microbiota. *Grifola frondosa* polysaccharide, for instance, has been shown to reduce blood glucose levels and increase the population of butyric acid-producing bacteria in diabetic mice (Ren et al., 2023). Similarly*, Agaricus bisporus* polysaccharides have been found to promote the growth of beneficial bacteria such as *Prevotella* and *Bacteroides* while also regulating the composition of the gut microbiome (Fu et al., 2023). Therefore, investigating the interaction between the gut microbiota and polysaccharides is crucial for elucidating the probiotic mechanisms at play.

This paper is focused on the digestive characteristics and effect of BAP on the gut microbiota. We systematically revealed the digestive and fermentation characteristics of BAP during digestion. To evaluate the fermentation process of BAP, fecal cultures were assayed for short-chain fatty acid (SCFA) production and assessed using 16S rRNA sequencing. Finally, by using an LPS-induced inflammatory cell model, the anti-inflammatory activity of metabolites produced by gut microbiota utilizing BAP was verified.

## 2 Materials and methods

### 2.1 Materials and chemicals

*B. aereus* was produced in Chuxiong City, Chuxiong Yi Autonomous Prefecture, Yunnan Province in March 2022. Pepsin, trypsin, α-amylase, and bile salts were purchased from Source Leaf Biotechnology Co. SCFA standards, including acetic acid, i-butyric acid, *n*-butyric acid, i-valeric acid and *n*-valeric acid, were purchased from Maclean Biochemical Co.

### 2.2 Preparation of BAP

To obtain *B. aereus* polysaccharides, ultrapure water was used to extract the powder twice at a ratio of 1:20 (*w*/*v*) at 80 °C for 4 h. Then, precipitation was achieved by adding 95 % (*v*/*v*) alcohol three times. Protein was separated by the Sevag method, and the resulting product was further processed via dialysis and freeze-drying. Subsequently, elution in a DEAE Sepharose fast flow column (26 mm × 300 mm) was performed to obtain BAP.

### 2.3 *In vitro* digestion

We conducted an *in vitro* digestion of BAP based on a prior study (Ma et al., 2022). Briefly, BAP (10 mg/mL) was mixed 1:1 (*v*/*v*) with simulated saliva (dissolved 0.15 g NaCl, 0.03 g CaCl_2_, 0.30 g KCl, and 30,000 U ɑ-amylase in 200 mL water) and regulated to 6.8. The samples were taken at 0.25 h, 0.5 h, and 1 h. The saliva sample (BAP-S) was blended with the simulated gastric fluid (dissolved 0.64 g NaCl, 0.03 g CaCl_2_, 0.22 g KCl, 0.12 g NaHCO_3,_ and 17,500 U pepsin in 200 mL water) at 1:1 (*v*/*v*), and the pH was regulated to 3. Various samples were collected at 0.5 h, 1 h, 2 h, 4 h, and 6 h. The gastric fluid sample (BAP-G) was blended with the intestinal fluid (1.08 g NaCl, 0.07 g CaCl_2_, 0.13 g KCl, 0.80 g pig bile salt, 3000 U pancreatin, and 50,000 U trypsin in 200 mL deionized water) at 10:3, and the mixture was regulated to pH 7.5. Various samples were collected at 0.5 h, 1 h, 2 h, 4 h, and 6 h.

### 2.4 Physicochemical property analysis of BAP

After simulated digestion, 3,5-dinitro salicylic acid was applied to determine the changes in reduced sugar content. The digestibility of BAP was calculated using the following formula:

$$Degree\;of\;hydrolysis\;\left(\%\right)=\frac{reducing\;sugar\;released}{total\;suger-initial\;reducing\;sugar}\;\times 100$$

Based on a prior study, with minor adjustments, the molecular weight and functional groups were determined (Jiang et al., 2021). The molecular weight of BAP was assayed by high-performance gel permeation chromatography (HPGPC).

Monosaccharide composition analysis of BAP was measured through GC–MS. Briefly, a solution of BAP (3 mg) was prepared by dissolving it in 1 mL of trifluoroacetic acid (2 mol/L) and heating at 90 °C for 6 h. After cooling to room temperature, the liquid was removed. Then, methanol (1 mL), hydroxylamine hydrochloride (100 μL) and pyridine (400 μL) were added, and the mixture was kept at 90 °C for 30 min. Then, 500 μL of acetic anhydride was added for 30 min at 90 °C, and 1 mL of methanol was added after blowing nitrogen. Monosaccharides were then detected by GC–MS. The carrier gas flow rate was 1 mL/min of ammonia, the shunt ratio was 50:1, the inlet temperature was 280 °C, and the column temperature was 200 °C.

For FT-IR analysis, BAP powder (1–2 mg) and KBr (200 mg) samples were prepared and put into an infrared spectrometer (Thermo Scientific Nicolet iS20, USA) for testing (Hu et al., 2023). BAP and its digestion components were coated with a gold film and then examined using a scanning electron microscope (SEM) system under high vacuum conditions. The surface morphological properties of BAP were observed by an SEM system at an accelerating potential of 5 kV.

### 2.5 Antioxidant activity assessment of digested BAP

The method employed previously was utilized to measure the ability of BAP to scavenge hydroxyl radicals and ABTS (Bai et al., 2023). Radical scavenging activity values were expressed as IC50 values (mg/ml), the concentration needed for 50 % radical inhibition by BAP or VC.

### 2.7 *In vitro* fermentation of BAP

An *in vitro* fermentation model was constructed using a previous study with modifications (Ma et al., 2022). In brief, we obtained fresh feces from healthy volunteers (2 males and 2 females). Equal amounts of fresh intermediate internal feces were scraped and thoroughly mixed. The mixture was then combined with sterile phosphate-buffered saline. After centrifuging the suspension at 2500 rpm and 4 °C for 5 min, the supernatant was obtained. The fecal supernatant was mixed with fermentation medium containing 1 % (*w*/*v*) BAP at a 1:9 (*v*/*v*) ratio and subsequently fermented at 37 °C for 48 h in an anaerobic incubator. In addition, no carbon source was added as the blank group, and oligosaccharide was added as the positive control group. The degree of fermentation in each group was calculated using the previous formula.

### 2.8 Measurement of reducing sugars, residual carbohydrates, and pH during *in vitro* fermentation

The reducing sugar content was assessed by the 3,5-dinitrosalicylic acid method, following the previously described method, at the conclusion of the *in vitro* fermentation. To measure the total carbohydrate content, the phenol–sulfuric acid method was employed, with glucose being utilized as a benchmark (Mecozzi, 2005). A pH meter was utilized to measure the pH at various time points, including 0 h, 6 h, 12 h, 24 h, and 48 h.

### 2.9 Analyses of gut microbiota

The fermentation broth was transferred to −80 °C for storage. The samples were then centrifuged to extract the precipitate for DNA extraction. The microbial composition of all DNA samples was examined through amplification of the V3-V4 region of 16S rRNA and sequencing on the BGISEQ platform. Tags were clustered to operational taxonomic units (OTUs) with USEARCH (v7.0.1090). Alpha diversity was analyzed by mothur (v.1.31.2) and measured by the Chao1 index, ACE index, Shannon index, Simpson index, etc. Beta diversity analysis was performed using QIIME software (v1.80). Microbial analysis was performed using R (v3.4.1) software with three samples per group.

### 2.10 Measurement of SCFAs

We determined the concentrations of SCFAs by employing gas chromatography with some slight modifications to a previously established method (Bai et al., 2023). First, the samples were acidified with 10 % sulfuric acid, extracted with ethyl acetate, and finally filtered through an organic filter membrane of 0.22 μm. The determination of SCFAs was carried out using gas chromatography with an Agilent 6890 N instrument, a DB Fast FAME column, and an FID detector.

### 2.11 Measurement of anti-inflammatory effects

The RAW 264.7 cell model was constructed based on a previous description (Bai et al., 2023). Under standard culture conditions, a dose of 100 μg/mL of 48 h fecal fermented liquid comprising blank, fructooligosaccharide (FOS), and BAP was applied to the culture system after 12 h of culture. After 2 h of incubation, LPS was added to the basal lateral compartment at 1 μg/mL to stimulate RAW 264.7 macrophages. The culture medium supernatant was collected, and IL-1β and TNF-α levels were determined by the Ruixin Biology ELISA kit, while NO levels were determined by the NO kit. Three separate experiments were conducted, each using three parallel wells.

### 2.12 Statistical analysis

The data from three independent experiments are presented as the means ± SDs. Correlation analysis and Mantel tests were performed using R software (4.2.3). Significance analysis was performed via the Duncan test, and *p* < 0.05 was considered significant. SPSS 22.0 software was used to conduct one-way ANOVA tests for intergroup comparisons.

## 3 Results and discussion

### 3.1 Change in BAP *in vitro* digestion

#### 3.1.1 Reducing sugar variation

**Fig. 1:**
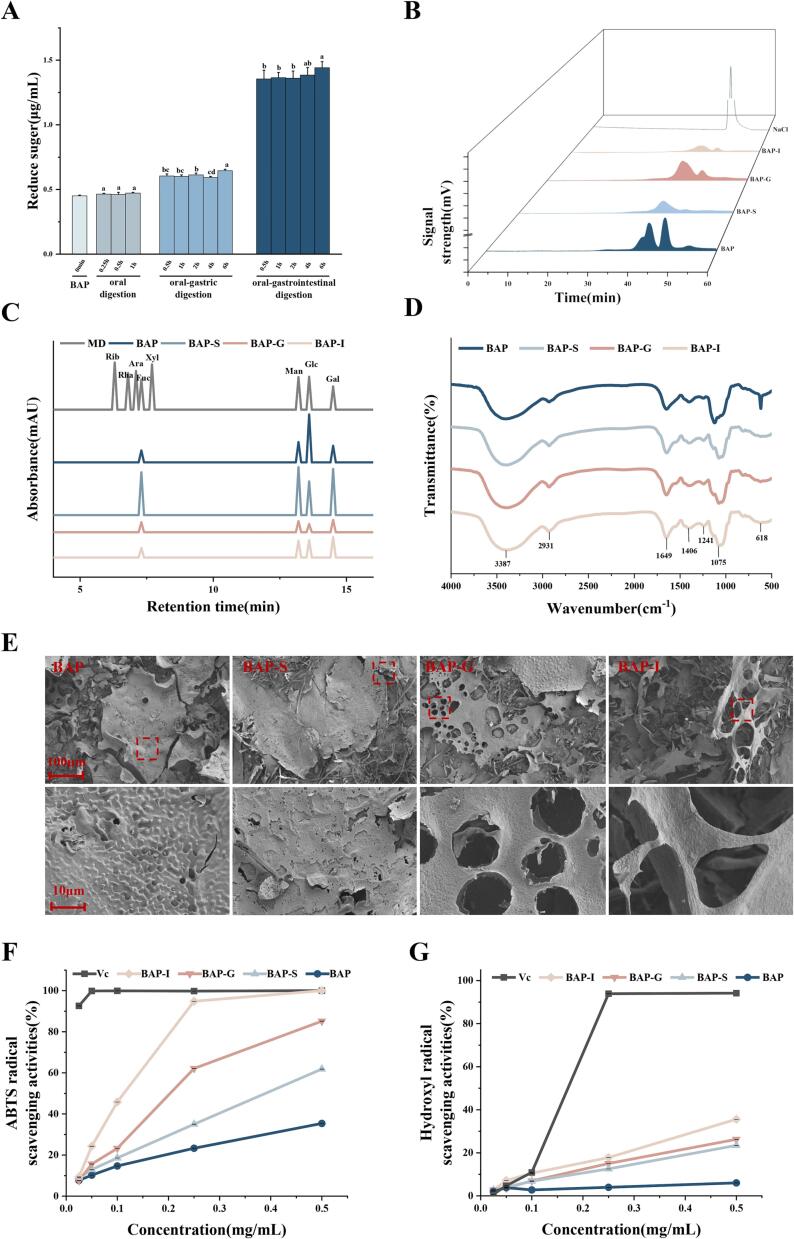
Changes in reducing sugar contents (A), HPGPC chromatograms (B), GC–MS of monosaccharide compositions (C), FT-IR spectra (D), SEM images (E) of BAPs during *in vitro* digestion. Changes in ABTS (E) and hydroxyl radical scavenging activities (F) of BAPs during digestion.

Fig. 1A illustrates the changes in BAP reducing sugar concentration throughout the simulated digestion process. The undigested BAP exhibited a reduced sugar concentration of 0.45 ± 0.01 mg/mL. The digestibility of BAP was 3.97 % during *in vitro* simulated digestion. Following salivary digestion, the reducing sugar concentration of BAP remained relatively unchanged at 0.47 ± 0.01 mg/mL, showing no significant difference. However, after undergoing simulated digestion in the gastrointestinal tract, the reducing sugar concentration of BAP increased to 0.65 ± 0.01 mg/mL and 1.44 ± 0.04 mg/mL. This increase in reduced sugar content of digested BAP indicated the breaking of glycosidic bonds.

#### 3.1.2 Changes in the structure of BAP during digestion

The molecular weight of polysaccharides and their functional groups are closely associated with their biological activity (K. Wang et al., 2019). Upon simulated digestion, the retention time of BAP exhibited an increase, as indicated by the rightward shift of the corresponding chromatographic peak, as presented in Table S1 and Fig. 1B. This shift suggested a slight decrease in the molecular weight (Mw) of BAP. The variation in the molecular weight of polysaccharides can be attributed to polysaccharide aggregation and the breaking of glycosidic bonds (Carnachan et al., 2012). In fact, polysaccharides usually accumulate and form aggregates in the water phase system. Studies have shown that the decrease in the Mw of polysaccharides from kiwifruit during digestion primarily occurs due to the disintegration of polysaccharide aggregates rather than the cleavage of glycosidic bonds (Carnachan et al., 2012). Considering the reducing sugar results, it can be inferred that the reduction in molecular weight of BAP primarily arose from the disintegration of aggregates and the partial breakage of glycosidic bonds within BAP.

The monosaccharide composition of polysaccharides is closely associated with their unique properties and structure (Liu et al., 2021). To assess the monosaccharide composition, GC–MS analysis was conducted. Fig. 1C illustrates that the major monosaccharide composition of BAP consisted of Gal (galactose), Glc (glucose), Man (mannose), and Fuc (fucose). The monosaccharide types remained unchanged following digestion with saliva, gastric juice, and intestinal juice. However, the proportions of monosaccharide composition in BAP, BAP-S, BAP-G, and BAP-I were found to differ (Table S2), indicating that *in vitro* digestion can influence the monosaccharide composition of polysaccharides. This effect was likely attributed to the actions of digestive enzymes such as pepsin, pancreatin, and trypsin, as well as the presence of HCl.

As shown in Fig. 1D, FT-IR spectroscopy was utilized to analyze the functional groups and structural features of BAP. Specifically, the hydroxyl group exhibited a stretching vibration at 3387 cm^−1,^ while the C—H bond experienced a stretching vibration at 2931 cm^−1^ (Wu, Yuan et al., 2021). The C=O bond caused absorption peaks at both 1649 cm^−1^ and 1406 cm^−1^ (L. Li et al., 2020), while the C—O—C bond contributed to the absorption peak at 1241 cm^−1^ (Wu, Fu et al., 2021). Additionally, the absorption peak at 1075 cm^−1^ was ascribed to the C—O—C stretching vibration and C—O—H bending vibration of the pyran ring (W. Wang et al., 2018). *In vitro* digestion has been found to have an insignificant effect on the functional groups of BAP, as indicated by the similar spectral structures observed in BAP-S, BAP-G, and BAP-I after digestion. The FT-IR spectra of *Volvariella volvacea* physicochemically showed similar structures, which was consistent with our results (Hu et al., 2023).

Undigested BAP exhibited a closely arranged and smooth surface (Fig. 1E). After digestion, noticeable changes in the morphological features of BAP were observed. Saliva digestion resulted in slight fragmentation, while digestion with gastric juice led to the appearance of fragments with smaller areas and holes on the surface. BAP-I displayed pores and a porous honeycomb structure on its surface. Previous studies have shown that acidic conditions in the stomach disrupt the complex internal structure of polysaccharides, leading to changes in their apparent form (Geng et al., 2023). These findings indicated that the digestion process significantly impacted the morphological features of BAP due to the partial breakdown of polysaccharides. The spatial structure of polysaccharides was closely linked to their biological activity; the presence of pores on the surface of the polysaccharide structure can increase its contact area, thereby enhancing its antioxidant activities (Cai et al., 2018). Based on speculation, the specific surface area of BAP-I was greater than that of BAP, primarily due to its porous surface. This porous structure exposed the effective structural domain of the polysaccharide, thereby improving its bioavailability.

In the *in vitro* digestion process, the surface structure of BAP exhibited holes and fragments under the catalysis of pepsin, pancreatin and trypsin. Previous studies have shown that the reduction in the molecular weight of polysaccharides was due to polysaccharide aggregation and glycosidic bond breakage (Carnachan et al., 2012). Moreover, the molecular weight of *Nostoc commune* Vauch polysaccharide decreased after simulated digestion *in vitro*, which was consistent with our results (Li et al., 2022). Therefore, the acidic environment during digestion will lead to the breaking of some glycosidic bonds and the destruction of aggregates, resulting in a slight reduction in the molecular weight of BAP and a slight change in the proportion of monosaccharides. These results show that the BAP structure is partially altered but generally stable under simulated digestive conditions *in vitro*. However, the human digestive system was affected by factors such as age and gender, so the consistency between *in vitro* simulation and *in vivo* digestion needs many subsequent verifications in the future.

#### 3.1.3 Changes in antioxidant activity during digestion

The antioxidant activity of polysaccharides is due to the attachment of electron donor or hydrogen donor functional groups to polysaccharide chains (Ahmad, 2021). The antioxidant ability of polysaccharides is influenced by various factors, including their chemical composition, morphological structure, and molecular weight (Ahmad, 2021). To assess the impact of *in vitro* digestion on the antioxidant ability of BAP, further investigation is necessary. Fig. 1F and G illustrate that both undigested and digested BAP exhibited significant ABTS and hydroxyl radical scavenging activity, with the scavenging ability being concentration dependent. The IC_50_ values of BAP-S, BAP-G and BAP-I for ABTS were 0.41, 0.23 and 0.12 mg/mL, respectively, and the scavenging ability of ABTS was 36 % at the highest concentration of BAP (0.5 mg/mL). Notably, BAP-I showed a significant increase in scavenging ability for ABTS and hydroxyl radicals. The primary reason for the improvement in antioxidant activity, according to studies, was the decrease in the molecular weight of *Nostoc commune* Vauch polysaccharide following digestion (Li et al., 2022). In addition, the contents of Ara, Gal and Man in the monosaccharide composition were found to be related to the antioxidant activity (Chen et al., 2019). Combined with the IC_50_ changes of BAP at different stages, we hypothesized that the structural changes of BAP during digestion affected its antioxidant activity. Specifically, the reduction in the molecular weight of BAP makes it easier to cross the membrane barrier and give full play to its biological activity. In addition, the pores on the surface of BAP increased the contact area after digestion, exposing its domain and enhancing antioxidant activity.

### 3.2 Change in BAP *in vitro* fermentation

#### 3.2.1 Changes in reducing sugar contents, total residual carbohydrates, and pH during *in vitro* fermentation

**Fig. 2:**
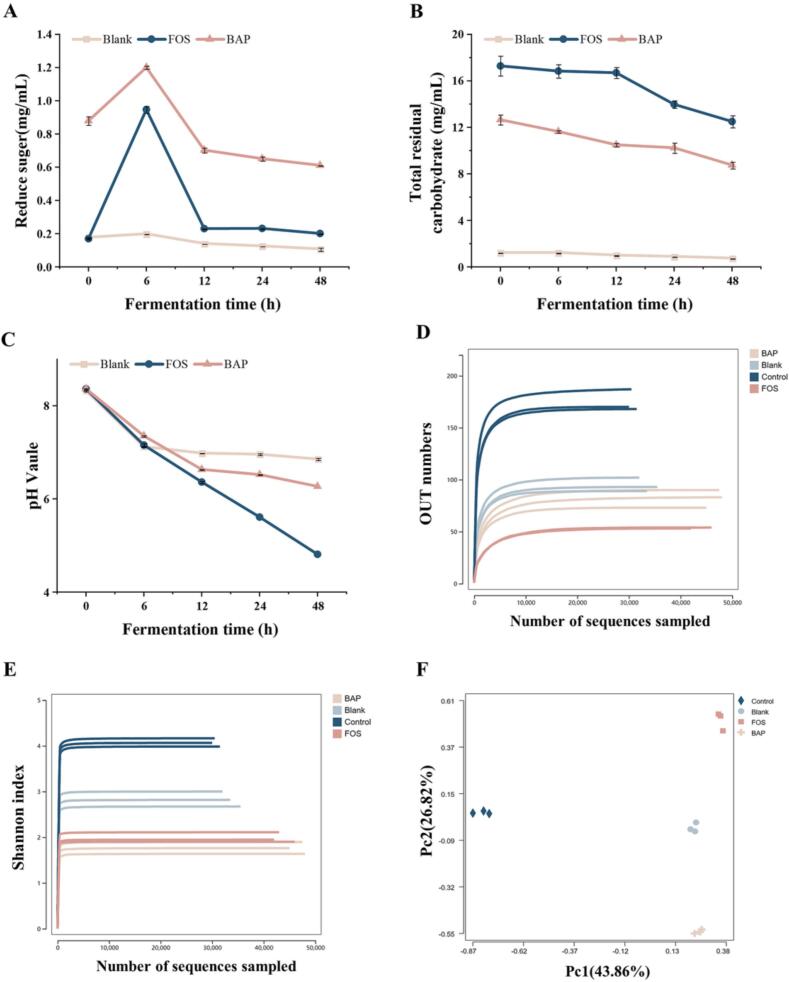
Changes in reducing sugar content (A), total residual carbohydrate content (B), and pH value (C) during *in vitro* fermentation. Alpha diversity analysis of gut microbiota. Rarefaction Curve (D), Shannon index curve (E). Beta diversity analysis of gut microbiota. Principal Component Analysis (F).

Based on the digestion results, BAP is not completely hydrolyzed by enzymatic digestion, indicating that it can be utilized by the gut microbiota. The gut microbiota has the ability to produce probiotic metabolites, such as SCFAs, by utilizing polysaccharides as substrates (Wu, Nie, et al., 2021). The extent of polysaccharide hydrolysis during fermentation is influenced by factors such as the monosaccharide composition and the structure of glycosidic bonds (Liu et al., 2021). Moreover, the utilization of polysaccharides by the gut microbiota brought about changes in the concentrations of reducing sugars and carbohydrates. The data presented in Fig. 2A and B demonstrated that the carbohydrate content of BAP consistently decreased, while the reducing sugar content exhibited an initial increase for the first 6 h, followed by a subsequent decline. The fermentation degrees of BAP and FOS were 2.07 % and 0.18 %, respectively. The findings implied that the gut microbiota consistently utilized BAP over time as fermentation proceeded. The gut microbiota utilized the reducing sugars generated through the hydrolysis of BAP by glucoside hydrolase enzymes. This process provided a continuous supply of carbon sources for the gut microbiota to utilize over time. Importantly, the utilization trend of BAP by the gut microbiota was similar to that observed with oligofructose, a well-studied prebiotic.

Fig. 2C illustrates the changes in pH during the fermentation process of BAP at different time intervals. Initially, there were no significant differences in pH among the three groups (*p* > 0.05). However, as the fermentation proceeded, a significant decrease in pH was observed (*p* < 0.05). The pH values of the blank, FOS, and BAP groups exhibited distinct changes. Specifically, the blank group showed an increase of 1.46 in pH, while the FOS and BAP groups displayed increases of 3.55 and 2.49, respectively, compared with the initial pH values. The decrease in pH observed during the fermentation process was attributed to the production of SCFAs by the gut microbiota (Yin et al., 2020). As polysaccharides such as BAP are fermented by the gut microbiota, SCFAs such as acetic, propionic, and butyric acids are generated, leading to a decrease in pH. This phenomenon has been observed in other studies involving the fermentation of polysaccharides, such as *Flammulina velutipes* polysaccharide fermentation, where the pH decreased from 6.8 to 3.1 with the production of SCFAs (Yin et al., 2020). Therefore, the decrease in pH observed in the fermentation of BAP suggested that the gut microbiota effectively degraded and utilized BAP to produce SCFAs and other metabolites, resulting in a lower pH environment.

#### 3.2.2 Effects of BAP on the gut microbiota

The gut microbiota can influence energy metabolism and enhance immune function, so it is an important factor in maintaining physical health (Sekirov et al., 2010). Studies have shown that polysaccharides affect host function by changing the structural composition and metabolic characteristics of the gut microbiota (Yin et al., 2020). In the study of the effect of BAP as a carbon source on the growth of the two strains of probiotics, BAP and FOS showed the same prebiotic effect (Fig. S1 and S2). Therefore, the effect of baps on the gut microbiota was further studied *in vivo*. Fig. 2 displays the rarefaction curves, Shannon indices, rank abundance curves, and principal component analysis (PCA) results. To ensure that the sample adequately represented the species diversity, a sparse curve analysis was performed, which assessed the adequacy of sample size and sequencing depth. The rarefaction curves demonstrated that the gut microbiota data obtained from the samples were reliable and reflective of the diversity and richness of the microbial community. The diversity of microbial communities was further evaluated using the Shannon index, which provides a measure of species richness and evenness. The α diversity of the gut microbiota decreased in the BAP and FOS groups (Fig. 2D and E), which may be because some bacteria can use polysaccharides to become enriched in the fermentation solution, leading to a decrease in the richness and diversity of the gut microbiota (Tan et al., 2023). A rank abundance curve was used to illustrate the uniformity and abundance of the sample species. (Fig. S3). Furthermore, β-diversity analysis, specifically the PCA plot (Fig. 2F), was employed to compare the diversity of species between different samples and assess their similarity. PCA revealed that the BAP and FOS groups displayed significant differences from the blank group, indicating distinct microbial community compositions. Overall, the α-diversity and β-diversity analyses, along with the PCA results, suggested that the sequencing volume and sample size were adequate, allowing for reliable assessment of the impact of BAP on the gut microbiota composition and diversity.

**Fig. 3:**
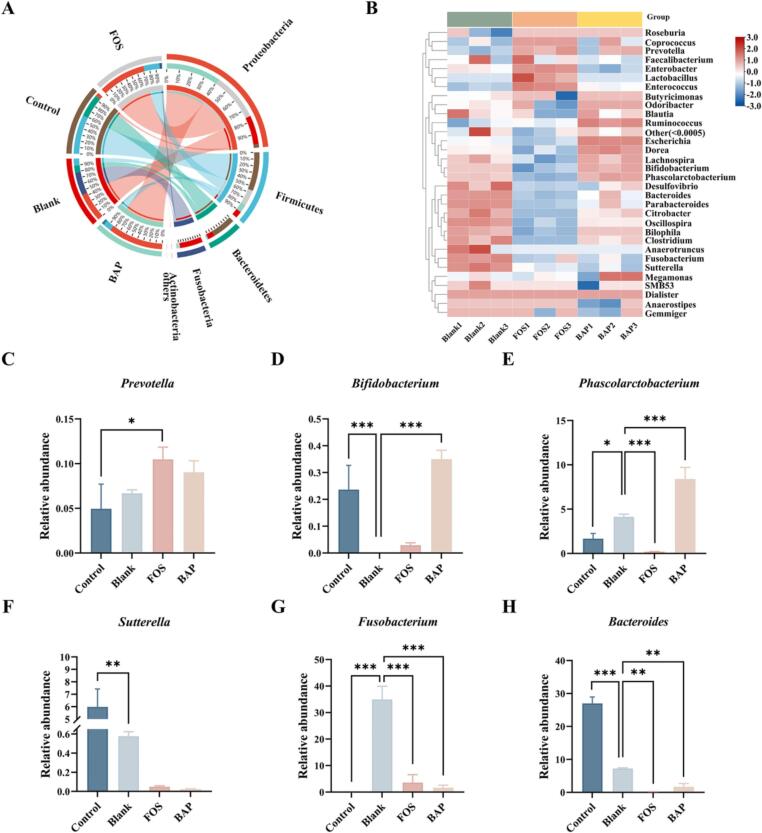
Effect of BAP on the gut microbiota composition. The relative abundance of gut microbiota at the phylum (A) level. Taxonomic heatmap of gut microbiota at the genus (B) level. Relative abundance of selected gut bacteria at the genus level (C—H).

At the phylum level, the feces control group was dominated by Firmicutes and Bacteroidetes bacteria. The blank group included Proteobacteria, Fusobacteria, Firmicutes, and Bacteroidetes, among which Proteobacteria and Fusobacteria exhibited an increase. The relative abundance of Proteobacteria and Firmicutes was increased, but Bacteroidetes and Fusobacteria decreased in the BAP- and FOS-treated groups (Fig. S4). Compared with the blank group, the ratio of Firmicutes to Bacteroidetes in the BAP and FOS groups was slightly higher. Moreover, the BAP and FOS groups had similar gut microbiota compositions. Firmicutes bacteria are known to possess carbohydrate-hydrolyzing enzymes that can metabolize polysaccharides into SCFAs, highlighting their role in polysaccharide metabolism (Geng et al., 2023). Clostridium, which belongs to the Firmicutes phylum, has been associated with mucous layers and has been classified as an opportunistic pathogen (Gupta & Sethi, 2014). BAP increased the population of bacteria that produce SCFAs, such as *Phascolarctobacterium*, *Prevotella*, *Bifidobacterium*, *Odoribacter*, *Bacteroides,* and *Coprococcus* (Fig. 3A and B). These bacteria have been associated with various beneficial effects. For instance, *Bifidobacterium* has been shown to upregulate AHR (aryl hydrocarbon receptor) and its target gene CYP1A1, which can alleviate colitis symptoms (Cui et al., 2023). *Phascolarctobacterium* has been found to reduce luminal succinic acid levels, restore colonization resistance, protect the colon mucosa from damage, and decrease the incidence of colitis in mice (Nagao-Kitamoto et al., 2020). On the other hand, the relative abundance of *Fusobacterium*, *Sutterella*, and *Bacteroides* was reduced in the BAP group (Fig. 3C-H). *Sutterella* has been associated with inflammation and has been implicated in causing ulcerative colitis (Kaakoush, 2020). *Bacteroides* has also been found to increase in patients with inflammatory bowel disease (IBD), leading to the worsening of colitis (He et al., 2022). These findings suggested that both BAP and FOS can modulate the composition of the gut microbiota by increasing the abundance of SCFA-producing bacteria and regulating gut homeostasis.

**Fig. 4:**
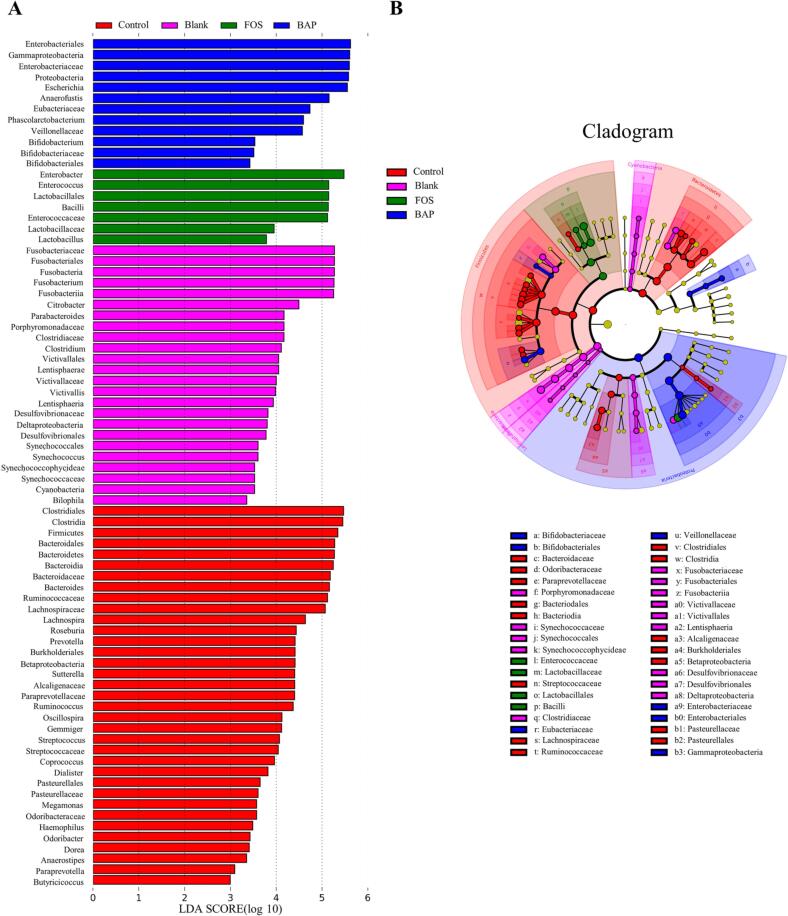
LEfSe analysis results. Histogram of LDA scores computed for differentially abundant features (A), Cladogram of LEfSe (B).

The LEfSe (linear discriminant analysis effect size) analysis revealed significant differences among all groups, with a total of 78 genera exhibiting LDA (linear discriminant analysis) scores above 2.0 (Fig. 4A). Among these genera, there were 12, 7, and 24 dominant genera in the BAP, FOS, and blank groups, respectively. The blank group was mainly composed of *Fusobacterium* and *Clostridium*. The FOS group is mainly composed of *Lactobacillus* and *Bacillus*. The BAP group was mainly composed of *Phascolarctobacterium* and *Bifidobacterium*. FOS and BAP could promote the growth of beneficial bacteria. This suggested that the composition of the gut microbiota was notably influenced by BAP and FOS supplementation. The evolutionary branching diagram is shown in Fig. 4B. *Bifidobacterium* and *Phascolarctobacterium* were found to be dominant species in the BAP group, which indicated that BAP could significantly adjust the microbial composition. These findings highlighted the potential of BAP to modulate the gut microbiota and promote the growth of specific beneficial genera, such as *Bifidobacterium* and *Phascolarctobacterium*, which could contribute to overall gut health.

#### 3.2.3 Effects of BAP on SCFAs

Studies have shown that SCFAs exhibit multiple benefits for the host, particularly in promoting the intestinal barrier and immunity (Ji et al., 2022). During fermentation, the total content of SCFAs showed an increase in the three groups (Table 1). After 48 h of fermentation, the BAP group exhibited a substantial increase in the total concentration of SCFAs, reaching 42.23 ± 0.95 mmol/L. The main SCFAs found in all groups were acetic acid, isobutyric acid, and *n*-valerate acid. The levels of butyric acid (7.71 ± 0.43 mmol/L) and isobutyric acid (7.10 ± 0.41 mmol/L) were observed to be higher in the BAP group than in the blank and FOS groups. Importantly, butyric acid was only present in the BAP group. The production of SCFAs is related to the utilization capacity of polysaccharides by some bacteria in the gut microbiota. Previous studies have shown a positive correlation between *Bifidobacterium* and butyric acid concentration. Therefore, the rise in the abundance of *Bifidobacterium* in the BAP group may be responsible for the elevation of butyric acid (Tan et al., 2023). On the other hand, studies have shown that polysaccharides containing galactose and mannose can promote the production of acetate and butyrate acids in the colon (Ding et al., 2017). Considering the monosaccharide composition of BAP, which included galactose and mannose, it was likely that these sugars contribute to the production of butyrate acids during fermentation. The regulation of metabolism and the modulation of cell differentiation and apoptosis are among the functions affected by butyric acid, a crucial energy source for colonic epithelial cells (Ziegler et al., 2016). Butyric acid has also been shown to alleviate diabetes in FFAR3-deficient mice (Lin et al., 2012). Additionally, the content of acetic acid in the BAP group and FOS group (19.06 ± 0.52 mmol/L and 36.95 ± 0.98 mmol/L) markedly exceeded that of the blank group (8.49 ± 0.52 mmol/L). In a study of tea polysaccharides, Bacteroides showed a negative correlation with almost all SCFA contents (Tan et al., 2023). The increase in acetic acid content in the BAP and FOS groups could be attributed to the decrease in Bacteroides abundance. Overall, the fermentation of BAP resulted in increased production of SCFAs, which have important physiological functions and potential health benefits. The presence of specific monosaccharides in BAP and the effects of BAP on certain gut microbiota may contribute to the observed changes in SCFA production.

**Table 1:** SCFA content at different time points during *in vitro* fermentation.

Groups	Time (h)	Acetic acid	i-Butyric acid	*n*-Butyric acid	i-Valeric acid	*n*-Valeric acid	Total SCFAs
blank	0	5.33 ± 0.34^bB^	3.84 ± 0.20^bB^	1.04 ± 0.35^aA^	0.72 ± 0.02^bA^	4.55 ± 0.06^dC^	15.47 ± 0.75^eB^
	6	5.44 ± 0.72^bB^	4.67 ± 0.48^aA^	ND	0.73 ± 0.00^abAB^	6.17 ± 0.21^cB^	17.00 ± 0.25 ^dB^
	12	7.80 ± 0.04^aC^	4.78 ± 0.13^aA^	ND	0.80 ± 0.08^abA^	7.83 ± 0.05^bA^	21.20 ± 0.19^cB^
	24	8.16 ± 0.52^aC^	4.47 ± 0.28^aB^	ND	0.87 ± 0.14^abA^	9.61 ± 0.22^aA^	23.12 ± 0.50^bC^
	48	8.49 ± 0.78^aC^	4.76 ± 0.43^aB^	ND	0.90 ± 0.03^aA^	10.67 ± 1.56^aA^	24.82 ± 0.51^aC^
FOS	0	5.88 ± 0.46^dAB^	4.92 ± 0.48^bA^	ND	0.63 ± 0.17^bA^	8.82 ± 0.47^cA^	20.25 ± 1.20^dA^
	6	6.41 ± 0.44^dAB^	4.78 ± 0.89^bA^	ND	0.64 ± 0.12^bB^	9.03 ± 0.48^cA^	20.86 ± 1.42^dA^
	12	18.97 ± 0.65^cA^	5.59 ± 0.27^abA^	ND	0.77 ± 0.17^abA^	9.13 ± 0.35^cA^	34.46 ± 0.74^cA^
	24	32.58 ± 0.63^bA^	6.15 ± 0.28^aA^	ND	0.92 ± 0.16^abA^	10.27 ± 0.18^bA^	49.91 ± 0.41^bA^
	48	36.95 ± 0.98^aA^	6.14 ± 0.72^aA^	ND	0.97 ± 0.20^aA^	12.00 ± 0.40^aA^	56.06 ± 0.92^aA^
BAP	0	6.47 ± 0.07^cA^	3.62 ± 0.18^bB^	ND	0.79 ± 0.05^bA^	5.29 ± 0.30^bB^	16.16 ± 0.19 ^dB^
	6	7.23 ± 0.25^cA^	4.07 ± 0.38^bA^	0.58 ± 0.14^bA^	0.82 ± 0.06^bA^	5.39 ± 0.80^bB^	18.10 ± 1.23 ^dB^
	12	14.52 ± 2.33^bB^	4.10 ± 0.73^bA^	0.71 ± 0.14^bA^	0.81 ± 0.10^bA^	5.28 ± 1.21^bA^	25.43 ± 2.34^cB^
	24	17.17 ± 1.36^aB^	4.20 ± 0.41^bB^	0.95 ± 0.24^bA^	0.81 ± 0.10^bA^	6.47 ± 0.36^abC^	29.60 ± 1.38^bB^
	48	19.06 ± 0.52^aB^	7.10 ± 0.41^aA^	7.71 ± 0.43^aA^	1.01 ± 0.05^aA^	7.36 ± 0.45^aB^	42.23 ± 0.95^aB^

blank: no additional carbon source supplement; FOS: FOS supplement; BAP: BAP supplement. Values represent the mean ± standard deviation, and different lowercase letters indicate significant differences among the same sample at different times (*p* < 0.05), while different capital letters indicate significant differences among the same time of different samples (*p* < 0.05). ND: not detected.

#### 3.2.4 Anti-inflammatory effect

The gut microbiota metabolites can affect the integrity of intestinal epithelial and mucosal barriers, immune response, and human microbiome diversity. To explore the impact of fermented BAP on inflammation, we assayed the concentration of inflammatory cytokines, including IL-1β, TNF-α, and NO, after intervention with fecal fermented liquid of blank (blank-F), FOS (FOS-F), and BAP (BAP-F).

**Fig. 5:**
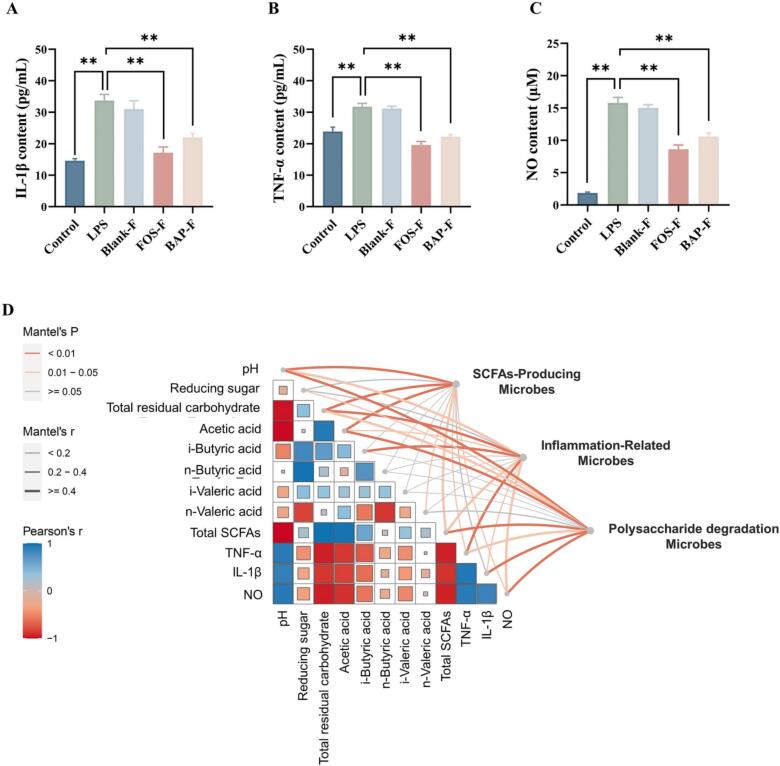
Metabolites of BAP utilized by the gut microbiota reduced the levels of proinflammatory cytokines in LPS-treated RAW264.7 macrophages (A-C). IL-1β (A) and TNF-α levels (B) and NO production (C) (*P < 0.05, **P < 0.001). Correlation analysis among the microbes, metabolites, and biochemical indices *in vitro* fermentation of BAP (D). Relationships among microbes, metabolites, and biochemical indices. The color gradient demonstrates Spearman’s correlation coefficients. Taxonomic (based on two independent indices: microbes and metabolites) and functional (based on biochemical indices) composition was related to inflammation by partial (geographic distance-corrected) Mantel tests.

The results showed that the concentration of inflammatory factors was significantly increased after LPS stimulation, confirming the successful establishment of LPS-stimulated inflammation (Fig. 5A-C). Treatment with BAP-F and FOS-F significantly decreased the concentrations of IL-1β, TNF-α, and NO (Fig. 5A-C). These findings indicate that the gut microbiota metabolites derived from fermented BAP can reduce the levels of inflammatory cytokines and potentially have alleviative effects on inflammation. Similar observations have been reported with fermented yam polysaccharide (CYP), where the fermented liquid (CYP-F) was more effective in reducing inflammatory factors compared to the polysaccharide alone (Bai et al., 2023). Currently, prebiotics are considered a beneficial approach to alleviate colitis by modulating the composition of the gut microbiota (H.-Y. Li et al., 2021). Furthermore, studies have shown that increasing the levels of butyrate and propionic acid in the intestine is an important mechanism through which *Lactobacillus acidophilus* alleviates inflammation (Arpaia et al., 2013). Based on these previous studies, it is speculated that the enhancement of anti-inflammatory activity in fermented BAP occurs through two main mechanisms. First, BAP can increase the abundance of beneficial bacteria and inhibit the growth of pathogenic bacteria, thereby improving the composition of the intestinal flora. Second, BAP can serve as a substrate for gut microbiota, leading to the production of metabolites such as SCFAs, which enhance anti-inflammatory activity through specific pathways. In summary, the findings suggest that fermented BAP has the potential to reduce inflammatory cytokine levels and exert anti-inflammatory effects. These effects may be attributed to changes in the gut microbiota composition and the production of metabolites, including SCFAs.

#### 3.2.5 Correlation analysis among microbes, metabolites, and other parameters *in the in vitro* fermentation of BAP

To further investigate the relationships among fermentation indicators, intestinal microbiome, SCFAs, and inflammatory cytokines, correlation analysis was performed (Fig. 5D). The correlation heatmap showed that pH during fermentation was negatively correlated with SCFAs, indicating that as pH decreases, the production of SCFAs increases. This finding aligns with previous experimental findings that the gut microbiota fermented and utilized polysaccharides, resulting in the production of SCFAs and a decrease in pH. Furthermore, the analysis revealed that the content of reducing sugars and total sugars positively correlated with SCFAs. This suggested that higher levels of reducing sugars and total sugars were associated with increased production of SCFAs. In terms of inflammatory cytokines, the concentrations of IL-1β, TNF-α, and NO showed a positive correlation with SCFAs; this suggested that higher levels of SCFAs may be associated with a decrease in the levels of inflammatory cytokines. This finding implied a potential anti-inflammatory effect of SCFAs, which was consistent with previous research. To further explore the relationships, the microbiota was divided into three types: SCFA-producing microbes, inflammation-related microbes, and polysaccharide degradation microbes. Mantel tests were conducted to assess the associations between these microbial groups and fermentation indicators, SCFAs, and inflammatory cytokines. Mantel tests showed that SCFA-producing microbes were related to pH (r = 0.797*p* = 0.001) and acetic acid (r = 0.893*p* = 0.001). Inflammation-related microbes were related to i-butyric acid (r = 0.659*p* = 0.006) and inflammatory cytokines (r = 0.638*p* = 0.006). Polysaccharide degradation Microbes were related to pH (r = 0.876*p* = 0.001) and total SCFAs (r = 0.819*p* = 0.001). These findings suggested that specific microbial groups played a role in the production of SCFAs, inflammation modulation, and polysaccharide degradation. The correlations observed highlighted the interplay among fermentation indicators, microbiome composition, SCFA production, and inflammatory cytokines.

## 4 Conclusions

In summary, in this study we investigated the digestive characteristics of BAP and its impact on gut microbiota. The results demonstrated that BAP underwent partial degradation during *in vitro* digestion, leading to significant changes in its physicochemical properties and biological activities. The fermentation of BAP by gut microbiota resulted in the consumption of carbohydrates and reducing sugars, accompanied by a decrease in pH. Importantly, specific gut microbes were identified as capable of utilizing BAP as a nutrient source and producing SCFAs, which helped modulate the surrounding gut environment. Furthermore, the metabolites produced by the gut microbiota utilizing BAP exhibited a remarkable inhibitory effect on the expression of the inflammatory cytokines IL-1β, TNF-α, and NO in RAW 264.7 cells stimulated by LPS. Overall, this study provides valuable insights into the digestive characteristics of BAP, its interaction with gut microbiota, and its potential anti-inflammatory properties. These findings contribute to the development and utilization of BAP for future applications in functional food products.

## CRediT authorship contribution statement

**Shixiang Wei:** Investigation, Formal analysis, Data curation, Visualization, Writing – original draft, Writing – review & editing. **Luanfeng Wang:** Data curation, Formal analysis, Resources. **Xiaodie Chen:** Investigation, Methodology. **Yue Wang:** Investigation, Methodology, Visualization. **Lingling Tong:** Data curation, Software. **Qianyun Han:** Project administration. **Bo Ren:** Project administration, Resources, Software, Supervision, Writing – original draft. **Dongsheng Guo:** Funding acquisition, Resources, Visualization.

## Declaration of Competing Interest

The authors declare that they have no known competing financial interests or personal relationships that could have appeared to influence the work reported in this paper.

## Data Availability

Data will be made available on request.
