# Supplementary material for: Anti-inflammatory activity of Boletus aereus polysaccharides: Involvement of digestion and gut microbiota fermentation
Source: Food Chem X. 2023 Dec 9;21:101052. doi: 10.1016/j.fochx.2023.101052 (PMC10770587; doi:10.1016/j.fochx.2023.101052)
Supplement: Supplementary data 1 [file mmc1.docx]

**Materials and methods**

**Effect of BAP on the growth of probiotics**

The growth of *Lactobacillus delbruckii* and *Lactobacillus reuteri* when BAP was used as a carbon source was measured with reference to the previous assay method (Wang, et al., 2022). *Lactobacillus delbruckii* and *Lactobacillus reuteri* were purchased from Shanghai Baiyi Biotechnology Co., LTD. MRS Culture-medium was purchased from Beijing Solaibao Technology Co., LTD. MRS Medium without glucose was supplemented with 2% BAP and FOS, respectively. The control group was MRS Medium without carbon source. The medium was inoculated with 4% Lactobacillus germani suspension and Lactobacillus delbruckii, respectively, and incubated at 37 ° C for 48 h. The growth of the two strains was monitored by the OD values at 600nm of the cultures in different time periods.

Wang, L., Lian, J., Zheng, Q., Wang, L., Wang, Y., & Yang, D. (2022). Composition analysis and prebiotics properties of polysaccharides extracted from Lepista sordida submerged cultivation mycelium. Front Microbiol, 13, 1077322.

**Tab. S1** The molecular weight distribution of BAP after simulated digestion

| **Samples** | Peak1 | | | Peak2 | | |
| --- | --- | --- | --- | --- | --- | --- |
|  | Mw（Da） | RT（min） | Area % | Mw（Da） | RT（min） | Area % |
| BAP | 26910 | 40.922 | 27.34 | 17729 | 42.441 | 67.53 |
| BAP-S | 24236 | 40.556 | 93.03 | ND | ND | ND |
| BAP-G | 22878 | 40.792 | 54.14 | 15079 | 41.497 | 39.90 |
| BAP-I | 22446 | 40.965 | 55.77 | 11664 | 41.862 | 40.56 |

ND: not detected.

**Tab. S2** Changes in the molar ratio of monosaccharides during in vitro digestion.

|  | BAP | BAP-S | BAP-G | BAP-I |
| --- | --- | --- | --- | --- |
| Fuc | 1 | 1 | 1 | 1 |
| Gal | 1.93 | 1.49 | 1.78 | 3.17 |
| Glc | 6.46 | 1.08 | 1.17 | 2.64 |
| Man | 2.61 | 1.69 | 1.75 | 2.79 |

ND: not detected.

In the in vitro growth test of probiotics, the strain in the control group barely grew due to the lack of carbon source (Fig. S1 and S2). Compared with MRS Group, BAP and FOS stimulated the growth of *Lactobacillus delbruckii* and *Lactobacillus reuteri* as carbon sources. In MRS group, FOS group and BAP group, the maximum absorbance of *Lactobacillus delbruckii* was 0.34, 0.47 and 0.41 (Fig. S1), and that of *Lactobacillus reuteri* was 0.57, 0.74 and 0.63 (Fig. S2). In BAP and FOS groups, *Lactobacillus delbruckii* began to grow rapidly at 12h, which was 4h earlier than that in MRS Group. This study demonstrates that BAP and FOS can promote the growth of *Lactobacillus delbruckii* and *Lactobacillus reuteri* in vitro and that BAP has the potential as a prebiotic.

**
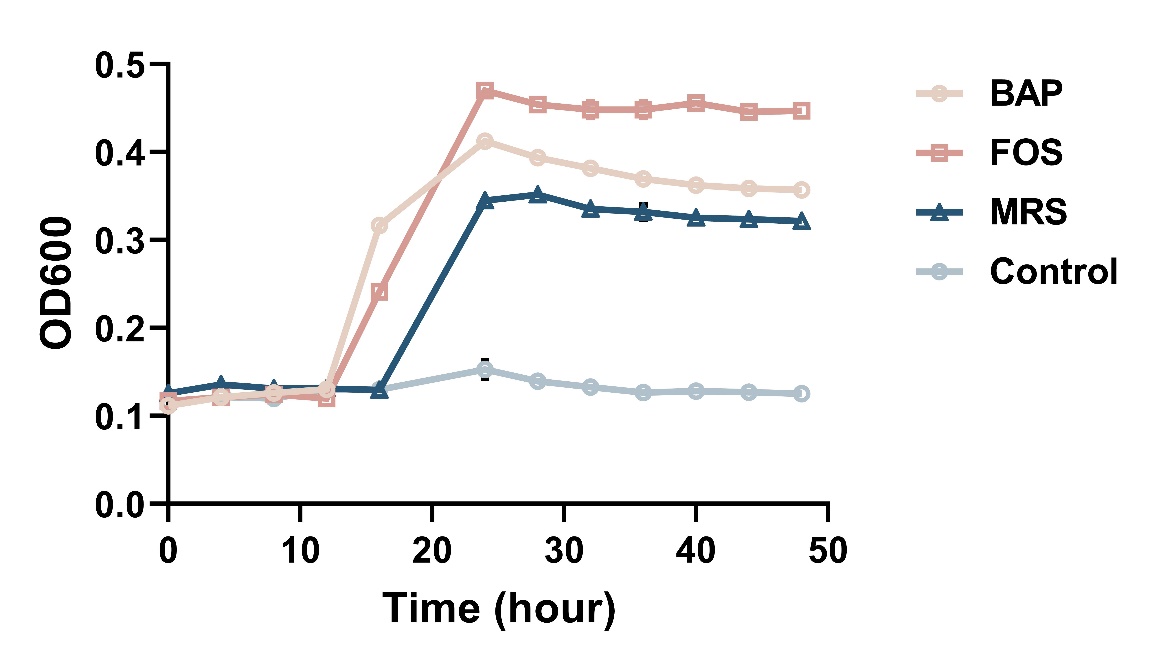
**

**Fig. S1** The growth curves for *Lactobacillus delbruckii* grown in basal MRS medium supplemented with various carbon sources

**
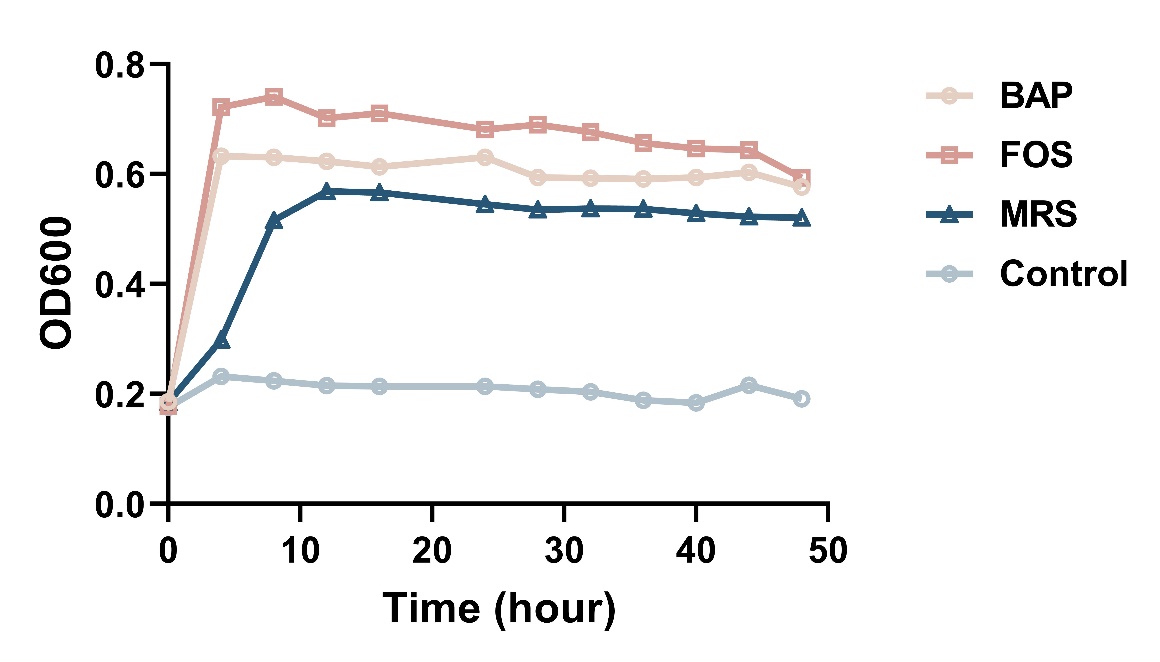
**

**Fig. S2** The growth curves for *Lactobacillus reuteri* grown in basal MRS medium supplemented with various carbon sources


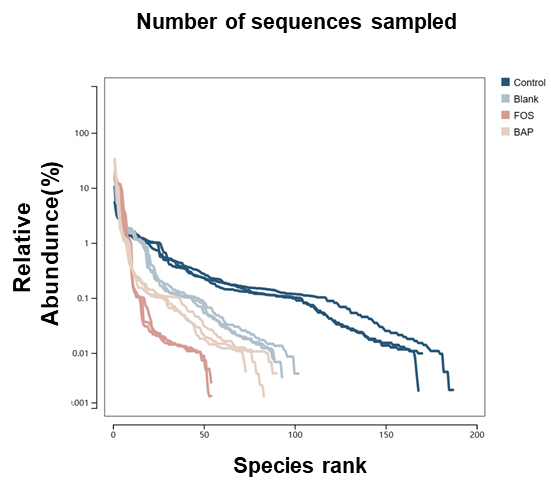


**Fig. S3** Rank abundance curve


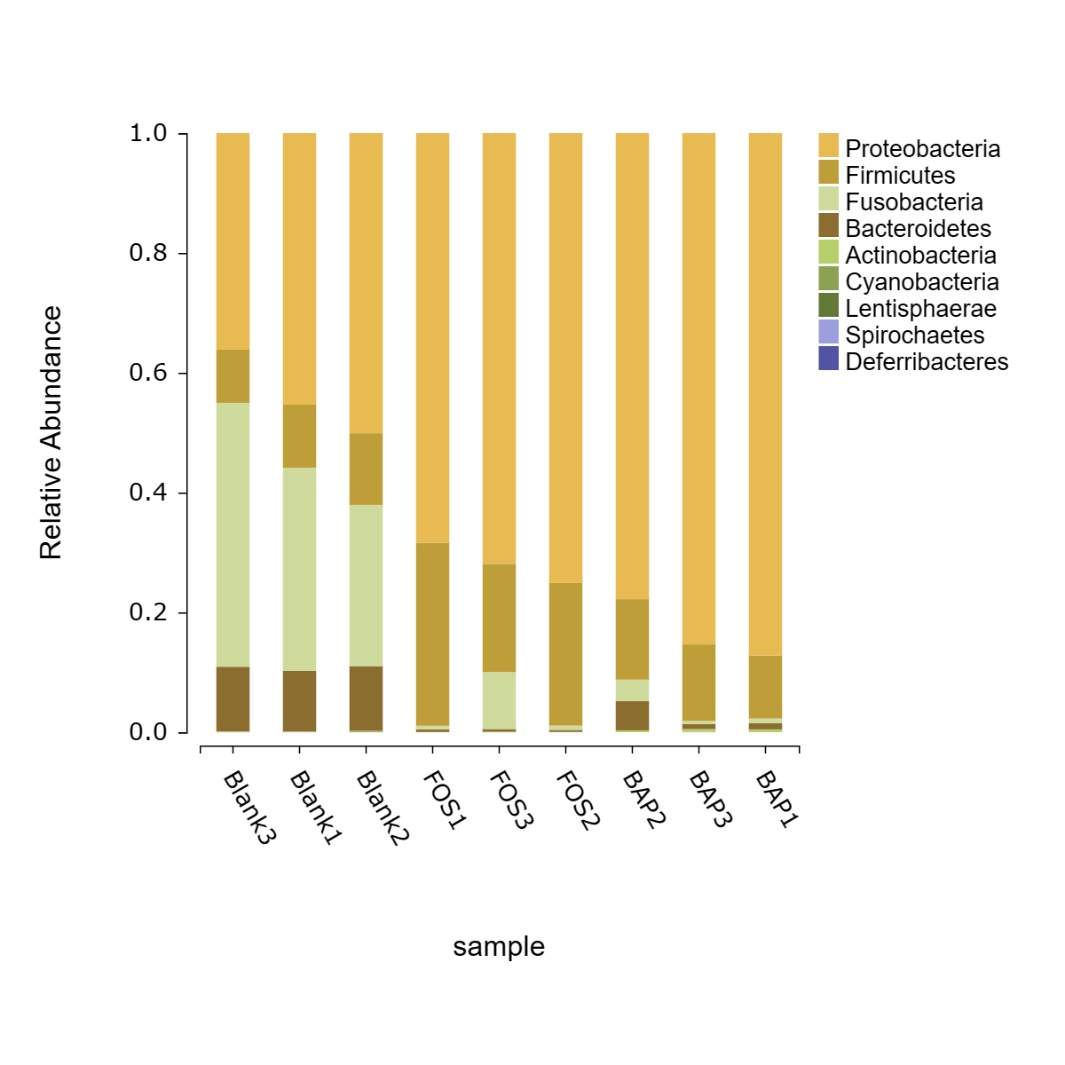


**Fig. S4** Effect of BAP on the gut microbiota composition. The relative abundance of gut microbiota at the phylum.
